# Combined modality PET/MR for the detection of severe large vessel vasculitis

**DOI:** 10.1186/s41824-022-00136-3

**Published:** 2022-08-15

**Authors:** John W. Cerne, Sophia Liu, Muhammad Umair, Ashitha Pathrose, Jackson E. Moore, Bradley D. Allen, Michael Markl, James C. Carr, Hatice Savas, Lisa Wilsbacher, Ryan Avery

**Affiliations:** 1 Department of Radiology, Northwestern University Feinberg School of Medicine, 420 East Superior St, Chicago, IL 60611 USA; 2 Biomedical Engineering, Northwestern University McCormick School of Engineering and Applied Science, Evanston, USA; 3 Department of Medicine, Northwestern University Feinberg School of Medicine, Chicago, USA

**Keywords:** PET, MRI, Inflammation, Vasculitis, Giant cell arteritis, Takayasu arteritis

## Abstract

**Background:**

Large vessel vasculitis (LVV) can be characterized based on symptom severity, and this characterization helps clinicians decide upon treatment approach. Our aim was to compare the imaging findings of combined modality positron emission tomography/magnetic resonance (PET/MR) and inflammatory markers between severe and non-severe LVV. A retrospective query was performed to identify all patients with LVV who underwent PET/MR at our institution between January 2015 and January 2021.

**Results:**

Eleven patients (nine females; age 62.2 ± 16.4 years) underwent 15 PET/MR scans. Positivity was defined by findings indicative of active LVV on each modality: PET positive if vessel metabolic activity > liver metabolic activity; MR positive if wall thickening or contrast enhancement. When positive PET or positive MR findings were considered a positive scan, LVV patients with severe disease (*n* = 9 scans) showed a higher number of positive scans (*n* = 9) compared to the number of positive scans in non-severe patients (*n* = 3) (*p* < 0.05). The sensitivity and specificity for the detection of severe LVV were 1.00 and 0.50, respectively. When only the presence of both positive PET and positive MR findings were considered a positive scan, inflammatory marker levels were not significantly different between severe and non-severe LVV groups (severe: erythrocyte sedimentation rate (ESR) = 9.8 ± 10.6 mm/h; C-reactive protein (CRP) = 0.6 ± 0.4 mg/dL) (non-severe: ESR = 14.3 ± 22.4 mm/h; CRP = 0.5 ± 0.6 mg/dL). Blood- and liver-normalized maximum standardized uptake values were not significantly different between severe and non-severe patients (1.4 ± 0.3 vs 1.5 ± 0.4; 1.1 ± 0.4 vs 1.0 ± 0.3, respectively).

**Conclusions:**

Because of the differences observed, PET/MR appears to be better suited to facilitate the characterization of LVV as severe or non-severe compared to inflammatory marker measurements and quantitative measurements of metabolic activity. Qualitative assessment of PET and MR positivity by ^18^F-fluorodeoxyglucose PET/MR may be able to supplement clinical symptoms-based LVV classification decisions and may be helpful when clinical symptoms overlap with other disease processes.

## Background

Large vessel vasculitis (LVV) is a noninfectious inflammatory disorder involving the aorta and its main branches. Giant cell (temporal) arteritis (GCA) and Takayasu arteritis (TA) represent the two major forms of LVV. The clinical manifestations of GCA can include headaches, scalp tenderness, jaw claudication, pulselessness, and limb claudication (Weyand and Goronzy 2014). TA more frequently occurs in younger women and presents with fatigue, upper extremity claudication, and headache (Sanchez-Alvarez et al. 2019). Given the overlapping clinical symptoms between LVV and other disease processes (Gulati and Bagga 2010) (hereditary diseases and fibromuscular dysplasia), serum biomarkers and conventional angiography have historically been used to support LVV diagnoses. However, inflammatory markers often do not correlate with LVV disease activity (Kerr et al. 1994; Tso et al. 2002), and conventional angiography cannot detect mural thickening and/or inflammatory changes that appear prior to stenosis (Gulati and Bagga 2010). Furthermore, this underscores the need for earlier detection of LVV to allow for earlier initiation of treatment prior to the development of stenotic vascular lesions.

MR angiography and ^18^F-fluorodeoxyglucose (FDG) positron emission tomography (PET) are increasingly utilized to noninvasively evaluate the vasculature of patients with suspected LVV because of their ability to detect inflammatory vessel changes that occur early in the disease process. MR angiography can show anatomic inflammatory signs such as intramural vessel wall edema, thickness, and contrast enhancement. ^18^F-FDG PET can show physiologic inflammatory signs, and have been shown to herald the development of complications such as aneurysm formation (Blockmans et al. 2008). The novel technology combined positron emission tomography (PET)/magnetic resonance (MR) has been introduced recently and PET/MR with ^18^F-FDG is gaining traction as a new modality for LVV evaluation, given its ability to simultaneously represent anatomic and physiologic tissue characterization under the same physiological conditions.

Once a diagnosis of active LVV is established, treatment strategies for LVV are decided upon based on the presence of life- or organ-threatening manifestations, which is categorized as severe LVV, or the lack thereof, which is termed non-severe LVV (Maz et al. 2021). It is recommended that severe GCA be treated with high-dose intravenous pulse glucocorticoids (GCs) followed by high-dose daily oral GCs, and that surgical intervention should be considered in cases of severe TA. In contrast, it is recommended that non-severe GCA be treated with only high-dose daily oral GCs and that non-severe TA should have continued treatment and monitoring (Maz et al. 2021). While ^18^F-FDG PET/MR findings and inflammatory marker measurements have been used to identify active LVV, they have not been used as basis to distinguish severe from non-severe LVV. There is a need to evaluate the imaging and laboratory measures associated with the subgroups of a symptoms-based LVV classification paradigm to understand if combined modality PET/MR has potential to supplement symptoms-based treatment decisions.

## Methods

### Subjects

A retrospective review was performed for the patients who underwent ^18^F-FDG PET/MR for clinically suspected LVV at our institution between January 2015 and January 2021. Each patient was reviewed in the electronic medical record (JWC) to classify subjects into either TA or GCA subgroups based on Sharma (Sharma et al. 1996) or American College of Rheumatology (Hunder et al. 1990) criteria, respectively.

### Clinical data

The electronic medical record was retrospectively reviewed to obtain the C-reactive protein (CRP) (Tina-Quant, Roche, Switzerland) and erythrocyte sedimentation rate (ESR) (Westergren method) values taken closest to the time of scan (< 2 months) (SL: a medical student with 1 year of clinical experience). Patients’ clinical histories were reviewed to determine classification as severe vs non-severe (JWC). If vision loss or ischemic symptoms were documented in the medical record, the patient was considered to have severe LVV (Maz et al. 2021).

### PET/MR acquisition

Patients fasted for at least 4 h before ^18^F-FDG injection and were required to have a pre-scan blood glucose level below 150 mg/dL in all subjects. After 60 min uptake time of FDG, patients underwent simultaneous PET/MR from skull base to upper thigh. Dixon method was used for attenuation correction. PET data was acquired between three to five minutes per each bed position while getting diagnostic MR sequences with either four or five total bed positions. PET/MR acquisition was performed on first-generation Biograph mMR (Siemens Medical Solutions, Erlangen, Germany), which enabled a simultaneous collection of PET and 3 Tesla (3 T)-MR images using a parallel imaging technique used primarily for 3D breath-hold abdominal imaging: “controlled aliasing in parallel imaging results in higher acceleration” sequence (CAIPIRINHA sequence) (field of view (FOV) 400 mm, voxel size 1.3 × 1.3 × 1.3 mm, repetition time/echo time (TR/TE) 650/22 ms, slice thickness 1.30 mm, frequency 320 Hz). Data were reconstructed iteratively by a 3D attenuation-weighted ordered subsets expectation maximization iterative reconstruction algorithm (3D OSEM) with three iterations and 21 subsets, Gaussian smoothing at 3.0-mm full-width at half-maximum and a zoom factor of 1.0. PET images were acquired with a matrix size of 344 × 344 × 127 voxels of 1.04 × 1.04 × 2.03 mm. Contrast medium (Gadobutrol, 1 mmol/mL) was injected intravenously at a standard dose of 0.2 mL/kg body weight.

#### Qualitative PET/MR assessment

**Fig. 1:**
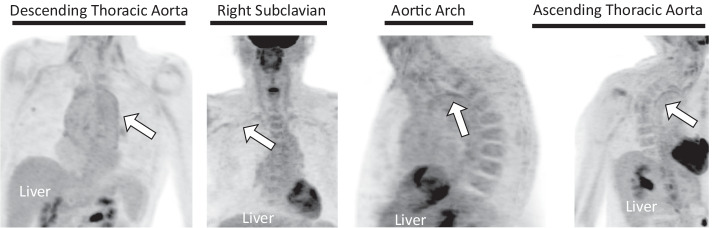
PET evaluation. PET grades on the PET portion of a patients’ PET/MR scans

**Fig. 2:**
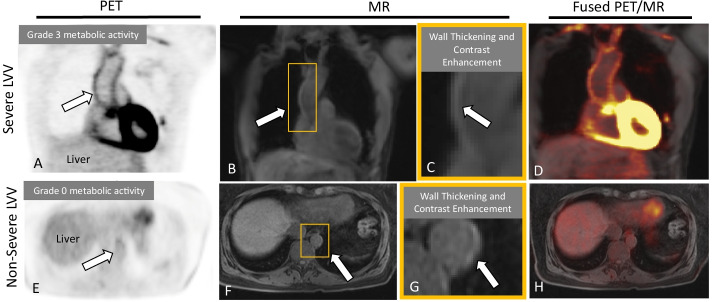
PET, MR, and PET/MR images. The MR, PET, and combined PET/MR findings in a patient with severe LVV (top row) presenting with a multi-year history of aching in bilateral legs and a stabbing pain in bilateral calves, and in a patient with non-severe LVV (bottom row), presenting with arthralgia and chronic constipation. Arrows point to an example vessel-of-interest in a severe LVV patient with positive PET and MR findings (at the ascending thoracic aorta) and in a non-severe LVV patient with positive MR findings (at the descending thoracic aorta). Positive MR findings more effectively ruled-in severe disease compared to PET, while negative PET findings more effectively ruled-out severe disease compared to MR

Scans were blindly assessed in a random order on a vessel-by-vessel basis, including the ascending thoracic aorta, aortic arch, descending thoracic aorta, suprarenal abdominal aorta, subclavian arteries, common carotid arteries, common iliac arteries, and femoral arteries (MU: a cardiothoracic radiology fellow with 5 years of experience in vascular imaging). Each of these vessels were examined for positive indicators of active LVV on PET (^18^F-FDG metabolic uptake in vessel walls greater than the uptake in the liver (Slart et al. 2018) (Fig. 1)) and on MR (wall thickening (Hartlage et al. 2014) and/or contrast enhancement (Küker et al. 2008) (Fig. 2)). The presence of any of these positive indicators were used to define each scan as PET positive or MR positive, respectively.

#### Quantitative PET assessment

Syngo.via (Siemens Medical Solutions) was used to create spherical volumes of interest (VOIs) on the most hypermetabolic region of every vessel (MU), within the right atrium blood pool (JWC), and within the dome of the right liver lobe^13^ (JWC). Care was taken to draw each circle so that the resulting right atrium and liver VOIs were between 2–3 cm^3^ and 3–4 cm^3^, respectively. The mean standardized uptake value (SUV_mean_) was obtained from each VOI. Blood- and liver-normalized SUV_mean_ values were calculated for each scan, using each scan’s highest vessel SUV_mean_ (Eqs. 1 and 2).

1 $$Blood\, Normalized \,{SUV}_{mean}=\frac{Vessel \,{SUV}_{mean}}{Blood \,{SUV}_{mean}}$$

2 $$Liver \,Normalized \,{SUV}_{mean}= \frac{Vessel \,{SUV}_{mean}}{Liver \,{SUV}_{mean}}$$

### Statistical analysis

Fisher’s exact test was used to compare the distribution of positive scans and the inflammatory marker measurements between groups. Independent samples T tests or Mann–Whitney U tests were performed based on normality testing by Shapiro–Wilk test. Significance definition: *p* < 0.05. Statistical analyses were carried out using SPSS v23 (IBM corporation, Armonk NY, USA).

## Results

### Subjects

**Fig. 3:**
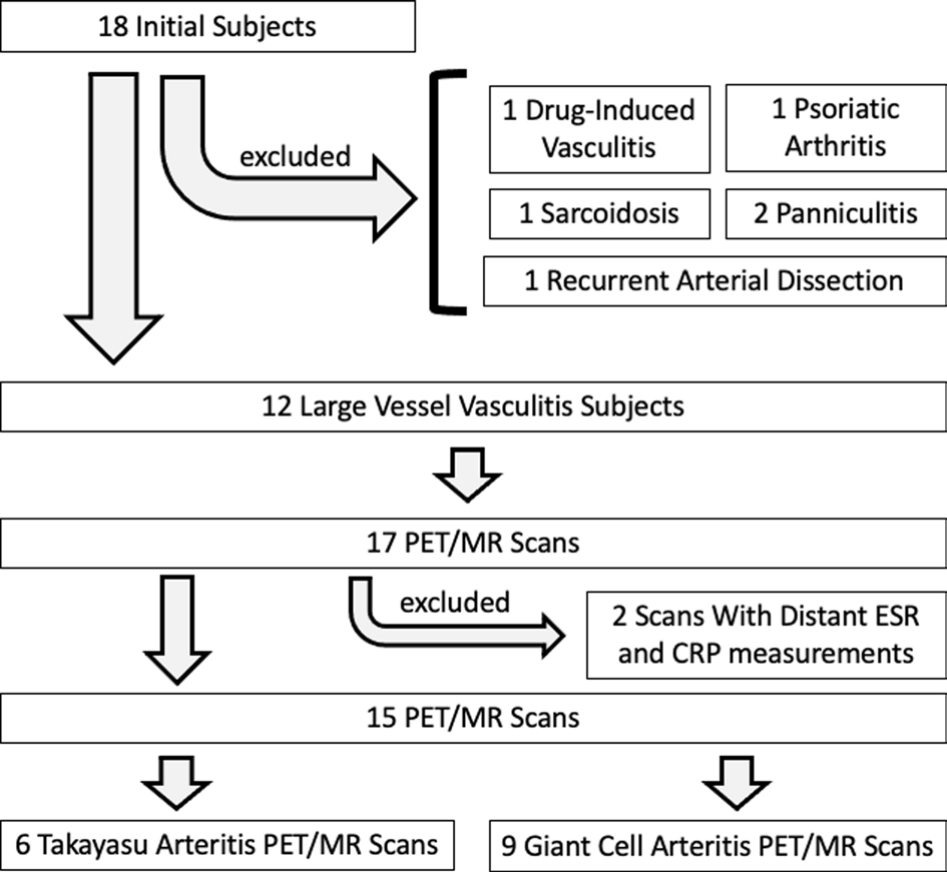
Exclusions. A flowchart of the excluded subjects in this study

The initial retrospective query yielded 18 unique subjects. Six subjects did not have large vessel vasculitis and were excluded. Of the resulting 17 PET/MR scans from LVV patients, two did not have inflammatory marker measurements taken near the time of scan (< 2 months) and were excluded (Fig. 3). This resulted in a final cohort size of 11 subjects, four of which had baseline and follow-up scans (demographic data in Table 1). Symptoms indicative of severe LVV were seen in six subjects (nine PET/MR scans), with documentation in the medical record occurring near the time of the PET/MR scan (32 ± 36 days).

**Table 1:** Demographic data: non-severe and severe LVV patients

	Non-severe LVV (*N* = 6)	Severe LVV (*N* = 9)	*p* value
LVV subtype			
Giant cell arteritis	5 (83%)	4 (44%)	0.29
Takayasu arteritis	1 (17%)	5 (56%)	0.29
Immunosuppression			
Steroids	1 (17%)	2 (22%)	1.00
Biologic	1 (17%)	2 (22%)	1.00
Steroid + Biologic	3 (50%)	1 (11%)	0.24
Atherosclerosis risk factors			
Smoking	3 (50%)	1 (11%)	0.24
Diabetes	0 (0%)	0 (0%)	1.00
Hypertension	2 (33%)	6 (67%)	0.31
BMI > 25 kg/m^2^	4 (67%)	2 (22%)	0.14
Hyperlipidemia	4 (67%)	5 (56%)	1.00
2 or more risk factors	4 (67%)	6 (67%)	1.00

*LVV* large vessel vasculitis, *BMI* body mass index

#### Laboratory inflammatory marker assessment

**Fig. 4:**
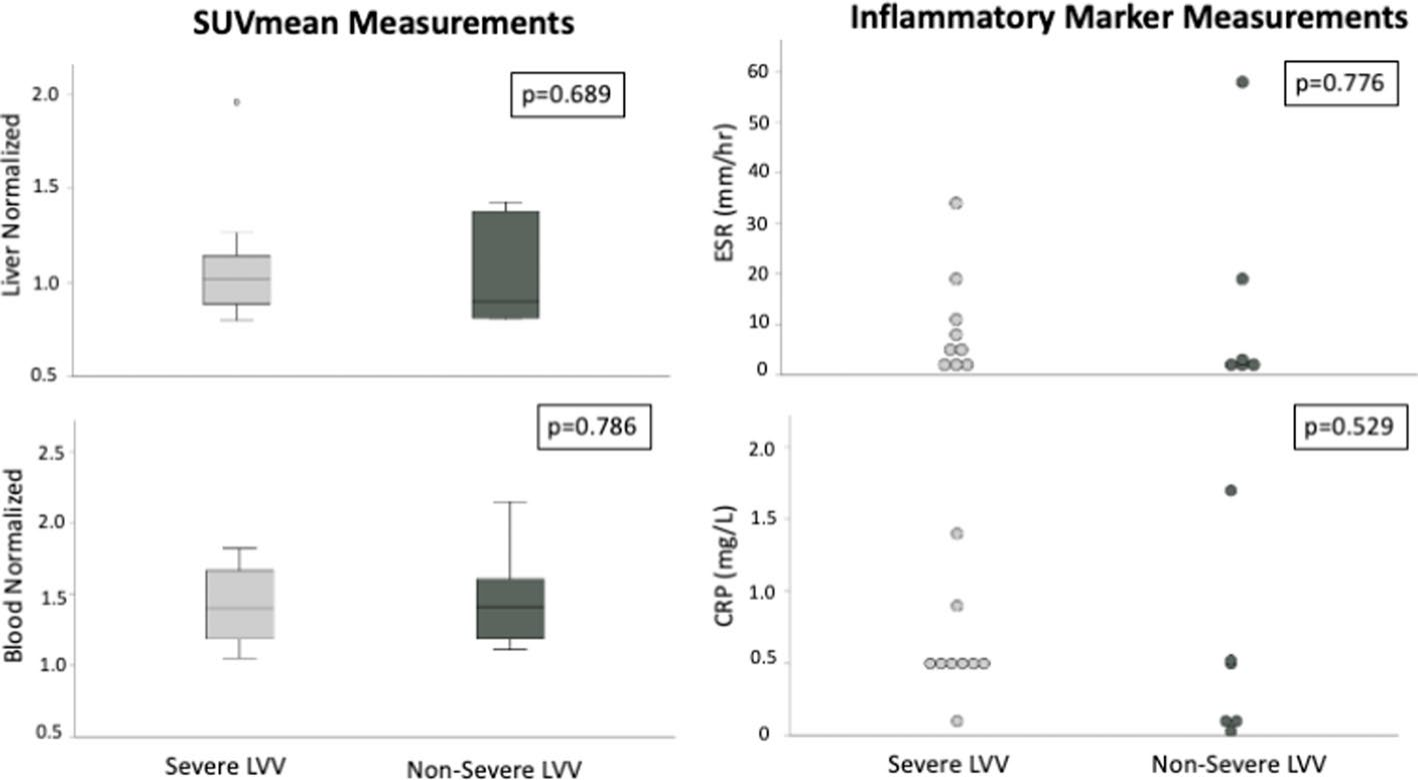
Inflammatory marker measurements. Box plot comparisons of SUVmean and inflammatory marker measurements between patients with severe and non-severe LVV

Inflammatory marker levels were not significantly different between severe and non-severe LVV groups (severe: ESR = 18 ± 17 mm/hr; CRP = 1.1 ± 0.8 mg/dL) (non-severe: ESR = 38 ± 41 mm/hr; CRP = 1.0 ± 1.0 mg/dL) (*p* values = 0.536 and 0.585, respectively) (Fig. 4).

#### Qualitative PET/MR assessment

**Fig. 5:**
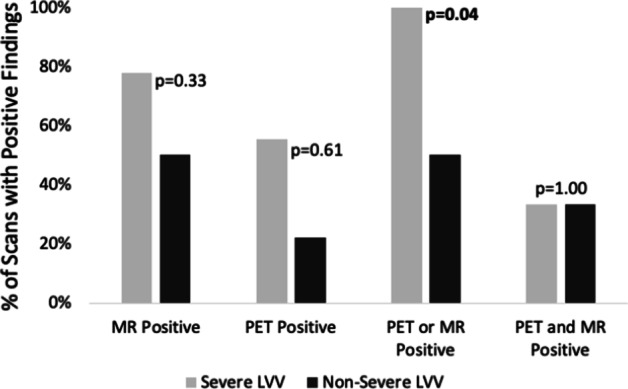
The percentage of scans with positive findings as defined by various positive definitions. MR was more sensitive than PET for the detection of vasculitis (severe or non-severe) (MR positive bars higher than PET positive bars). When positive PET or MR findings were considered to represent a positive scan, positive scans were more often found in severe LVV than in non-severe LVV

**Fig. 6:**
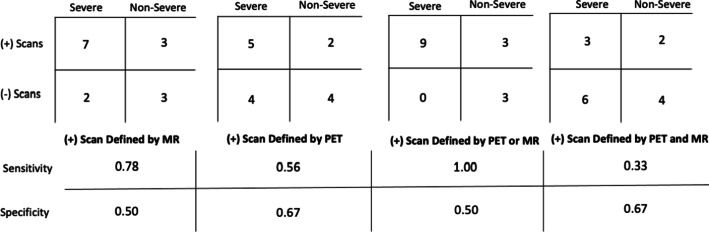
The sensitivities and specificities associated with various positive definitions. The associated sensitivities and specificities for the identification of severe large vessel vasculitis, when positivity was defined by [MR], [PET], [PET or MR], or [PET and MR]. When positivity was defined by [PET or MR], the sensitivity for the detection of severe large vessel vasculitis was the highest. Symptomatic measures were used for ground-truth LVV classifications

Smaller branch vessels from the aorta were difficult to evaluate due to the low spatial resolution of the PET scanner, and definitive PET scoring was unable to be performed for the right subclavian artery in 8/15 scans, left subclavian artery in 4/15 scans, right common carotid artery in 7/15 scans, left common carotid artery in 4/15 scans, right common iliac artery in 4/15 scans, left common iliac artery in 4/15 scans, right femoral artery in 6/15 scans, and left femoral artery in 6/15 scans. Low spatial resolution also prevented MR grading of the left femoral artery in 1/15 scans, right femoral artery in 1/15 scans, and left common carotid artery in 1/15 scans. The combined use of PET and MR was able to detect severe LVV compared to non-severe LVV (*p* = 0.04), more effectively than either modality in isolation (*p* > 0.05) (Fig. 5). When positive scan thresholds were defined by [positive MR findings; positive PET findings; positive PET or MR findings; positive PET and MR findings], the sensitivities and specificities for the detection of severe LVV were [0.78 and 0.50; 0.56 and 0.67; 1.00 and 0.50; 0.33 and 0.67, respectively] (Fig. 6).

#### Quantitative PET assessment

Blood (VOI size = 2.46 ± 0.32) and liver (VOI size = 3.50 ± 0.29)-normalized SUV_mean_ values were not significantly different between severe and non-severe patients (1.4 ± 0.3 vs 1.5 ± 0.4, *p* = 0.786; 1.1 ± 0.4 vs 1.0 ± 0.3, *p* = 0.689, respectively) (Fig. 4).

## Discussion

This retrospective exploratory proof of concept study used LVV disease severity as a clinical reference to assess the utility of integrated PET/MR in patients with LVV. The examined clinical subgroups of LVV in this study were associated with statistically different findings on PET/MR; however, inflammatory markers and quantitative PET measurements showed no statistically significant difference between patient subgroups. The findings of our study broadly agree with the findings in other studies, yet direct comparisons are complicated by varied LVV classification approaches.

In this study, qualitative evaluation of positive findings with either PET or MR when read in conjunction on the fused PET/MR was able to distinguish severe from non-severe LVV, while a combined consideration of positive PET and MR findings was unable to distinguish these groups. Using a distinct LVV classification method, Laurent et al. used a review of simultaneous PET and MR positivity to define an inflammatory pattern (Laurent et al. 2019), and found that an inflammatory pattern was associated with active disease in both GCA (as defined by the presence of clinical signs and increased CRP level (> 10 mg/L)) and TA (as defined by a National Institute of Health stroke score > 2). Although an “inflammatory pattern” was identically characterized in our study [PET positive and MR positive], we did not find a statistically significant difference in our clinically defined groups. In our study, the added consideration of isolated PET positive findings was able to identify a statistically different distinction between severe and non-severe LVV. LVV clinical status has been shown to correlate with PET (*p* < 0.01) and not magnetic resonance (*p* = 0.70) in a previous study by Quinn et al., which further supports the added benefit of PET imaging in LVV patients (Quinn et al. 2018). In cases where the spatial resolution of MR is too low, PET activity may be able to provide a new basis on which to identify inflammatory changes, yet this application may be inappropriate for the examination of smaller vessels. There are certain physical limitations to PET imaging which create a fundamental limit for improvements in spatial resolution. PET spatial resolution is largely restricted by the size of the detector element, positron range, acollinearity, decoding, penetration, and sampling error. Given these barriers, practical PET cameras can only be made with up to 2.36 mm full width at half-maximum spatial resolution (Moses 2011). Attempting to detect radiotracer uptake in target volumes of a few cubic millimeters has been suggested as a flawed application of PET imaging (Alavi et al. 2017). Using high-frequency ultrasound, Svensson et al. determined that the intima media thickness of the common femoral and subclavian arteries was 0.49 ± 0.11 and 0.53 ± 0.13 mm, respectively (Svensson et al. 2022), which reinforces our inability to localize PET uptake in many of these vessels. With PET, vasculitis-associated large-vessel inflammation detection may be most appropriately used to detect thickened vessels or inflamed aortic foci. Future work should compare PET and MR findings under various MR field strengths and with improved PET resolution to see if there is a higher association between the two, on a vessel-by-vessel basis.

In our study, the trends and values of biomarkers did not show statistically significant differences between severe and non-severe LVV subgroups. Biomarkers have previously been studied with reference to vasculitis disease activity (Kerr et al. 1994; Tso et al. 2002; Rodriguez-Pla et al. 2020) and in with reference to PET imaging findings (Walter et al. 2005). Tissue inhibitor of metalloproteinase-1 (TIMP-1), ESR, and B cell-attracting chemokine 1 (BCA)-1/CXC motif ligand 13 (CXCL 13) were shown to be higher in active GCA than GCA in remission when disease activity was assessed by physician’s global assessment of disease activity (0–10 scale) (Rodriguez-Pla et al. 2020). In contrast, Kerr et al. and Tso et al. found poor correlations between inflammatory markers and disease activity in LVV (Kerr et al. 1994; Tso et al. 2002). In a study by Walter et al., patients’ grades of LVV uptake (grades I-III) were positively correlated with ESR (*p* = 0.007) and CRP (*p* = 0.002) (Walter et al. 2005). Our preliminary findings suggest that biomarkers may not be able to distinguish clinical LVV subtypes, but future studies with larger cohorts are warranted.

For each scan’s maximum blood- and liver-normalized SUV_mean_ value, no statistically significant difference was found between severe and non-severe LVV. In a study by Laurent et al., it was shown that the median (of all observed vessels) SUV_max_ value was higher in LVV patients with PET positive and MR positive scans (i.e., an inflammatory pattern) compared to isolated MR positive scans (i.e., fibrous pattern) or normal scans (Laurent et al. 2019). In contrast to the study by Laurent et al., our study tested quantitative measurements when qualitative measurements did not necessarily indicate higher metabolic activity. We used quantitative PET SUV_max_ to assess the maximum uptake to test the hypothesis that severe LVV patients show higher metabolic activity in the most metabolically active vessels compared to non-severe LVV patients. To our knowledge, prior studies have not compared quantitative metabolic activity measurements in patient groups that may not have necessarily had higher amounts of qualitatively-assessed metabolic activity. In our study, because the qualitative PET findings were also not significantly different between clinical subgroups, we have confidence that our PET assessment techniques were valid. Though we did not find significant quantitative differences between our patient subgroups, we believe that more studies of this kind are warranted for research purposes.

### PET/MR alternatives

Alternative imaging techniques may still be appropriate means to evaluate LVV. It appears that the benefit of combined modality PET/MR stems from its ability to allow multi-modality comparison. Several false-positive pitfalls emerge when a single modality is used to evaluate LVV, such as an atheromatous plaque leading to PET uptake (Rudd et al. 2002). Vascular inflammation is best supported by the presence of several imaging findings, and this multimodality corroboration of LVV disease status can be obtained with an isolated modality, such as PET or MR, or a combined modality, such as PET/CT] (Lee et al. 2016). Although simultaneous PET/MR acquisitions facilitate the co-localization of vasculitis-associated lesions, [combined modality PET/CT] and [isolated PET and isolated MR] offer this same benefit though with slightly more radiation exposure and patient inconvenience.

## Limitations

Firstly, this exploratory proof of concept study is limited by the number of vessels that were unable to be assessed. Each scan had different vessels excluded from analysis. For this reason, the number of positive vessels in each scan were not evaluated and only the highest vessel SUV_mean_ was used for calculations. Similarly, a global summary of qualitative PET activity was not used and scans were qualitatively considered PET positive if there was at least one area with arterial ^18^F-FDG uptake higher than liver ^18^F-FDG uptake. Although an average measurement could have been used to describe the ^18^F-FDG uptake present in each scan (as SUV_artery_ measurements have been previously derived from several SUV_max_ territory measurements (Dashora et al. 2022)), we sought to singularly use the vessels with the highest ^18^F-FDG uptake to qualitatively define PET positive scans (binary variable) and quantitatively investigate the vessel in each scan with the most ^18^F-FDG uptake after normalization to the blood pool and liver (continuous variable). It follows that several PET-negative and MR-negative patients may represent false-negative findings, as low spatial resolution may have limited positive detection. PET and MR may therefore have higher sensitivity for the detection of severe large vessel vasculitis and/or lower specificity for the detection of non-severe large vessel vasculitis. Second, this study included a small number of patients due to the rarity of the disease and novelty of the modality. Yet with a cohort size of 11 subjects (15 scans), this study has similar power to a previously published study on this topic (Laurent et al. 2019, 18 scans). Third, ten PET/MR scans were taken while patients were receiving immunosuppression treatment (steroids, three scans; biologics, three scans; steroids and biologics, four scans). Immunosuppressive drugs can lead to a limited diagnostic accuracy of PET in LVV (Fuchs et al. 2012). In clinical practice, LVV patients are typically on immunosuppression at the time of PET/MR. Because our severe and non-severe groups did not display a statistically significant difference in immunosuppression use, we believe this to be less of a confounding factor and more of a basis to support the translation of our findings to patients with and without immunosuppression. Additionally, this was a retrospective review of the medical record, which may represent an imprecise way to realize clinical symptomatology. Often severe symptoms were noted, but the PET/MR scan occurred several weeks after a clinician’s note documented those severe symptoms were present in a patient. To mitigate this potential source of error and maintain consistency, a patient was ascribed to have severe LVV if there was any indication of the presence of severe symptoms regardless of temporal proximity to time of PET/MR. This retrospective study design also precluded the recording of inflammatory marker measurements on the same day of the PET/MR scan. Although ESR can take several weeks to normalize (Litao and Kamat 2014; Shusterman et al. 1985), the associations between patients’ scans, symptoms, and biomarkers may not be a true representative and hence a possible source of error. Further, the separation of LVV patients into (solely) severe and non-severe phenotypes may be an oversimplification of the clinical spectrum of disease present in the cohort. Several management recommendations put forth by the American College of Rheumatology use detailed LVV descriptors such as suspected disease, relapse, and remission as a basis to recommend LVV evaluation and treatment strategies (Maz et al. 2021). A previous study by Rimland et al. showed that inflammatory marker measurements and PET activity change at key moments in LVV disease course, such as the transition between remission and active disease status (Rimland et al. 2020). We initially attempted to classify patients using this more granular approach, but retrospective review of the medical record did not offer strong evidence to support specific disease categorizations. Prospective studies are needed, so that controlled definitions of disease status can be made at the time of a patient’s PET/MR scan. We believe that this is best defined by the physician who has been longitudinally following a patient, rather than third party inference from the electronic medical record.

## Conclusions

Qualitative assessment of PET and MR imaging biomarkers utilizing ^18^F-FDG PET/MR appear to supplement clinical symptoms based LVV classification decisions. This approach is promising for helping to differentiate severe from non-severe LVV when either positive findings were detected on either PET or MR. Inflammatory marker measurements, as well as quantitative measurements of PET SUV, appear less promising for differentiating severe from non-severe LVV.

## Data Availability

The datasets used and/or analyzed during the current study are available from the corresponding author on reasonable request.

## Abbreviations

LVV: Large vessel vasculitis
GCA: Giant cell arteritis
TA: Takayasu arteritis
PET: Positron emission tomography
CRP: C-reactive protein
ESR: Erythrocyte sedimentation rate
SUV_max_: Maximum standardized uptake value
